# The relationship between internalized stigma and quality of life among people with mental illness: are self-esteem and sense of coherence sequential mediators?

**DOI:** 10.1007/s11136-017-1596-3

**Published:** 2017-05-22

**Authors:** Piotr Świtaj, Paweł Grygiel, Anna Chrostek, Izabela Nowak, Jacek Wciórka, Marta Anczewska

**Affiliations:** 1 First Department of Psychiatry, Institute of Psychiatry and Neurology, Sobieskiego 9, 02-957 Warsaw, Poland; 2 Educational Research Institute, Górczewska 8, 01-180 Warsaw, Poland

**Keywords:** Internalized stigma, Quality of life, Self-esteem, Sense of coherence, Mental illness

## Abstract

**Purpose:**

To elucidate the mechanism through which internalized stigma reduces the quality of life (QoL) of people with mental illness by exploring the mediating roles of self-esteem and sense of coherence (SOC).

**Methods:**

A cross-sectional analysis of 229 patients diagnosed with schizophrenia or affective disorders was undertaken to test a sequential mediation model assuming that more severe internalized stigma is related to lower self-esteem, which is associated with weaker SOC, which in turn relates to worse QoL.

**Results:**

The proposed model was supported by the data. A sequential indirect effect from internalized stigma to QoL via self-esteem and SOC turned out to be significant [beta = −0.06, SE = 0.02; 95% CI (−0.11, −0.03)]. Support was also found for simple mediation models with either self-esteem or SOC as single mediators between internalized stigma and QoL.

**Conclusions:**

Self-esteem and SOC are personal resources that should be considered as potential targets of interventions aiming to prevent the harmful consequences of internalized stigma for the QoL of people receiving psychiatric treatment.

## Introduction

People with mental illness are frequent targets of public stigma, i.e., negative stereotypes, prejudice, and discrimination [1]. Many individuals turn these stigmatizing societal attitudes against themselves, which may result in internalized stigma (also referred to as self-stigma). Internalized stigma can be defined as *“a subjective process, embedded within a socio*-*cultural context, which may be characterized by negative feelings (about self), maladaptive behaviour, identity transformation, or stereotype endorsement resulting from an individual’s experiences, perceptions, or anticipation of negative social reactions on the basis of their mental illness”* [2, p. 2151]. This detrimental process may hamper the recovery of service users, limit their life chances and substantially reduce their quality of life (QoL) [1, 3].

However, although the negative impact of the internalized stigma of mental illness on QoL is well established [2, 4], the precise mechanism of this effect has not been fully explained. Some previous research has indicated that self-esteem may play a mediating role in this relationship. More specifically, in a study including 179 people with serious mental illness, Mashiach-Eizenberg et al. [5] found that self-esteem fully mediated the relation between internalized stigma and hope, whereas hope partially mediated the relationship between self-esteem and QoL. In another study, based on data obtained from 403 persons with mental illness, Oliveira et al. [6] demonstrated that self-esteem fully mediated the relation between internalized stigma and the physical and the social relationships domains of QoL, and partially mediated the relationship between internalized stigma and psychological, environment, and level of independence domains.

Building and expanding on these findings, we propose a model of the impact of internalized stigma on QoL including sense of coherence (SOC) as another potential mediating factor, in addition to self-esteem. SOC is the term coined by Antonovsky, who defined it as *“*a global orientation that expresses the extent to which one has a pervasive, enduring though dynamic feeling of confidence that (1) the stimuli deriving from one’s internal and external environments in the course of living are structured, predictable, and explicable; (2) the resources are available to one to meet the demands posed by these stimuli; and (3) these demands are challenges, worthy of investment and engagement*”* [7, p. 19]. These three interrelated components of SOC are called comprehensibility, manageability and meaningfulness. The SOC construct is the core of Antonovsky’s theory of salutogenesis, which seeks to explain the origins of health. The theory posits that high SOC reflects a greater capacity to cope with stressful situations and predicts good health and QoL.

Importantly, according to Antonovsky the development of a strong SOC requires the presence of so-called generalized resistance resources (GRRs). These include *“*any characteristic of the person, the group, or the environment that can facilitate effective tension management*”* [8, p. 99]. In other words, within the salutogenic framework, GRRs are biological, material, or psychosocial factors that lead to life experiences which contribute to seeing the world as more comprehensible, manageable, and meaningful.

Self-esteem is regarded as one of the typical GRRs promoting SOC [9]. This view was supported empirically by the studies performed on adolescent [10], adult [11], and elderly [12] populations, which revealed that better self-esteem predicted higher SOC. Hence, given that both cross-sectional and longitudinal research [2] consistently demonstrate a negative association of internalized stigma of mental illness with self-esteem, it seems reasonable to presume that self-esteem may mediate the relationship between internalized stigma and SOC. Furthermore, since there is a vast body of empirical evidence from studies on various samples (mainly disease-specific groups of patients) showing the positive influence of SOC on QOL [13], one can expect that SOC may act as a mediator between self-esteem and QoL.

Against this background, in the current study, we tested a theoretical model which assumed that self-esteem and SOC sequentially mediate the relationship between internalized stigma and QoL among people with mental illnesses. Specifically, we hypothesized that more severe internalized stigma is related to lower self-esteem, which is associated with weaker SOC, which in turn relates to worse QoL.

## Methods

### Sample

Study participants were recruited from various mental health care facilities of the Institute of Psychiatry and Neurology (IPN) in Warsaw (Poland). IPN is a large scientific research and clinical center providing a broad range of mental health services, mainly to the population of Warsaw and its environs. The inclusion criteria were as follows: (1) diagnosis of schizophrenia (F20) or affective disorders (F30-F33) according to the *International Classification of Diseases, 10th Revision* (ICD-10); (2) age over 18 years; and (3) a stable mental condition, according to the treating psychiatrist, sufficient to enable the understanding and accurate answering of the questions in the questionnaires. Individuals with active drug or alcohol dependence, organic brain disease, severe cognitive deficits, or documented mental retardation were excluded.

Of the 281 persons who were asked to participate in the study, 229 (81.5%) agreed and formed the study sample. Their socio-demographic and clinical characteristics are shown in Table 1.

**Table 1:** Socio-demographic and clinical characteristics of the participants (*n* = 229)

Characteristic	*n* (%) mean (SD)
Sex	
Women	142 (62.0)
Men	87 (38.0)
Age in years	45.90 (15.13)
Education	
Primary or vocational	37 (16.2)
Secondary	94 (41.0)
Higher	98 (42.8)
Marital status	
Married or cohabiting	76 (33.2)
Single (never married)	101 (44.1)
Widowed	12 (5.2)
Separated or divorced	40 (17.5)
Living situation	
Living with someone	173 (75.5)
Living alone	56 (24.5)
Employment status	
Employed	89 (38.9)
Unemployed	140 (61.1)
Illness duration in years	15.36 (12.00)
Type of psychiatric setting	
Inpatient ward	130 (56.8)
Day ward	17 (7.4)
Outpatient clinic	62 (27.1)
Community mental health center	20 (8.7)
Diagnosis (ICD-10 code)	
Schizophrenia (F20)	123 (53.7)
Bipolar disorder (F31)	56 (24.5)
Depressive episode (F32)	12 (5.2)
Recurrent depressive disorder (F33)	38 (16.6)

### Measures

Internalized stigma was evaluated with the use of the Stigma Experiences Scale (SES) from the Inventory of Stigmatizing Experiences (ISE) [14]. The SES is a complex measure encompassing several related subdomains (perceived stigma, experienced stigma, social withdrawal, and impact of stigma) [15]. This self-report questionnaire comprises 10 items which use different response formats. Following the procedure recommended by the instrument developers [14], the responses were recoded into binary variables: 0 = the absence of stigma and 1 = the presence of stigma. The index was created by summing up scores across all items. A higher total score indicates more severe internalized stigma. In this study, Cronbach’s alpha coefficient for the SES was 0.81.

QoL was measured with the extended version of the Satisfaction with Life Domains Scale (SLDS) [16]. This instrument includes 20 items assessing the level of satisfaction with various life areas and with life in general. Responses are made on a scale from 1 (worst QoL) to 7 (best QoL). In order to reduce the number of missing values, the overall score was calculated by summing up individual item scores and dividing the total by the number of valid answers. The higher the rating, the better the QoL. Cronbach’s alpha for the SLDS turned out to be 0.93.

Self-esteem was evaluated using the Rosenberg Self-Esteem Scale (RSES) [17]. This is a 10-item self-administered questionnaire employing a four-point response scale (1 = strongly agree; 4 = strongly disagree). All item scores were summed up and divided by the number of valid responses. A greater total score denotes higher self-esteem. The RSES demonstrated good internal consistency in our data (Cronbach’s alpha = 0.88).

SOC was assessed by means of the 29-item Sense of Coherence Scale (SOC-29) [7, 18]. This tool contains 11 comprehensibility, 10 manageability, and 8 meaningfulness items. Respondents are asked to select a response on a seven-point semantic differential scale with two anchoring phrases. In accordance with the intention of Antonovsky, who regarded the SOC-29 as a measure of a global orientation to life and saw no basis for deriving distinguishable subscores for comprehensibility, manageability, and meaningfulness [18], only a total scale score was utilized in the analyses. To calculate it, the responses for each item were summed and divided by the number of valid answers. A higher total score represents stronger SOC. In the current sample, the value of Cronbach’s alpha for the SOC-29 was 0.91.

The severity of psychopathological symptoms was measured with the standard version of the Brief Psychiatric Rating Scale (BPRS) [19]. This consists of 18 items scored by a clinician on a scale ranging from 1 (symptom not present) to 7 (symptom extremely severe). To create a global score, the sum of the item scores was divided by the number of valid items. The higher the score, the more severe the individual’s psychopathology. Cronbach’s alpha of the BPRS was found to be 0.91.

### Procedures

Ethical approval for the study was granted by the Bioethical Committee at the IPN. All participants provided their informed consent. The measures were administered by a trained clinician; however, the patients could also fill in the self-report questionnaires personally if they volunteered to do so. At the beginning, the participants answered a set of questions regarding their socio-demographic and clinical characteristics (where necessary, the information was supplemented or verified by reviewing their medical records). Next, the self-report scales were completed in the following order: the SES, SOC-29, RSES, and SLDS. Finally, psychopathology was assessed by a clinician with the use of the BPRS.

### Data analysis

Means and standard deviations or percentages, as appropriate, for all study variables, Cronbach’s alpha coefficients for the instruments used, and Pearson product-moment correlations between the key variables were computed by means of IBM SPSS Statistics version 23 (SPSS Inc., Chicago, IL).

**Fig. 1:**
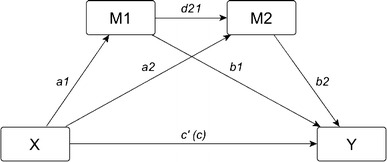
Schematic representation of a sequential mediation path model linking *X* to *Y* through *M1* and *M2*. *Y* is the dependent variable, *X* is the independent variable, and *M1* and *M2* are the two mediators; *a1* direct effect of *X* on *M1*, *a2* direct effect of *X* on *M2*, *b1* direct effect of *M1* on *Y*, *b2* direct effect of *M2* on *Y*, *d21* direct effect of *M1* on *M2*, *c′* direct effect of *X* on *Y*, *c* total effect of *X* on *Y*

In order to investigate whether self-esteem and SOC sequentially mediate the relationship between internalized stigma and QoL, we used a three-path sequential multiple mediational model. In such a model, two mediators intervene in a series between an independent and a dependent variable [20], as depicted in Fig. 1. Apart from the two-mediator chain, we also examined two simple mediation paths with either self-esteem or SOC as single mediators between internalized stigma and QoL.

To estimate the model, three regression equations were calculated: (1) regressing mediator 1 (self-esteem) on the independent variable (internalized stigma), (2) regressing mediator 2 (SOC) on mediator 1 (self-esteem) and the independent variable (internalized stigma), and (3) regressing the dependent variable (QoL) on mediator 1 (self-esteem), mediator 2 (SOC) and the independent variable (internalized stigma). In all three regression equations, the following socio-demographic and clinical variables were included as covariates: sex, age, education, marital status, living situation, employment, illness duration, type of psychiatric setting, diagnosis, and severity of psychopathological symptoms (BPRS total score). The size of the three specific indirect effects was compared using pairwise contrasts.

The hypothesized mediation model was analyzed by means of the PROCESS macro for SPSS [21], based on ordinary least-squares (OLS) regression (Model 6 as described in PROCESS). Since the PROCESS macro produces unstandardized coefficients, prior to analysis all continuous variables were standardized to clarify the interpretation and comparison of parameter estimates. The bootstrapping procedure recommended by Preacher and Hayes [22] was applied for testing the significance of the indirect effects. Unlike traditional tests, such as the Sobel test [23], bootstrapping does not require the assumption that the sampling distribution of the indirect effect is normal, which is difficult to meet for small research samples especially. We used 20,000 bootstrap resamples to calculate the bias-corrected 95% confidence interval (CI). If the interval does not include zero, the effect is statistically significant at *p* < 0.05.

## Results

Descriptive statistics and intercorrelations of the measures used in the study are presented in Table 2. Internalized stigma, QoL, self-esteem, and SOC were all significantly correlated in the expected direction, whereas psychiatric symptoms showed significant (positive) association only with internalized stigma.

**Table 2:** Descriptive statistics and intercorrelations of the measures used in the study (*n* = 229)

Measure	Mean (SD)	Range	1	2	3	4
1. Stigma Experiences Scale (SES)	3.58 (2.85)	0–10	–			
2. Satisfaction with Life Domains Scale (SLDS)	4.46 (1.05)	1–7	−0.31**	–		
3. Rosenberg Self-Esteem Scale (RSES)	2.70 (0.55)	1–4	−0.29**	0.61**	–	
4. Sense of Coherence Scale (SOC-29)	4.31 (0.97)	1–7	−0.36**	0.62**	0.70**	–
5. Brief Psychiatric Rating Scale (BPRS)	1.59 (0.61)	1–7	0.18**	−0.05	0.02	−0.07

For all instruments, higher scores indicate higher levels of the measured constructs

*SD* standard deviation

* *p* < 0.05, ** *p* < 0.01

**Fig. 2:**
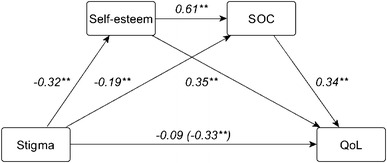
Sequential mediation path model with self-esteem and SOC as mediators in the relationship between internalized stigma and QoL (*n* = 229). In parenthesis: total effect of internalized stigma on QoL; before parenthesis: direct effect of internalized stigma on QoL. Covariate paths were estimated, but are not reported in the figure. *p < 0.05, **p < 0.01

The results of the mediation analysis are shown in Fig. 2 and in Tables 3 and 4.

**Table 3:** Results of the regression analyses testing the sequential mediation effect of self-esteem and SOC in the relationship between internalized stigma and QoL (*n* = 229)

Predictor	Direct effect (SE)			Total effect (SE)
	Self-esteem	SOC	QoL	QoL
Constant	0.00 (0.16)	0.15 (0.13)	0.01 (0.13)	0.06 (0.16)
Internalized stigma	−0.32 (0.07)**	−0.19 (0.06)**	−0.09 (0.06)	−0.33 (0.07)**
Self-esteem	–	0.61 (0.05)**	0.35 (0.08)**	–
SOC	–	–	0.34 (0.09)**	–
Sex^a^	0.00 (0.15)	0.00 (0.11)	0.01 (0.12)	0.01 (0.15)
Age	−0.06 (0.10)	0.18 (0.07)**	−0.07 (0.09)	−0.04 (0.11)
Education^b^				
Primary or vocational	0.17 (0.20)	0.19 (0.18)	0.18 (0.18)	0.34 (0.23)
Higher	0.21 (0.17)	0.22 (0.11)*	−0.10 (0.12)	0.09 (0.14)
Marital status^c^	0.20 (0.18)	−0.10 (0.12)	0.03 (0.13)	0.11 (0.16)
Living situation^d^	0.06 (0.20)	0.04 (0.12)	−0.03 (0.13)	0.01 (0.16)
Employment^e^	−0.04 (0.17)	0.09 (0.11)	−0.02 (0.11)	−0.01 (0.14)
Illness duration	0.01 (0.08)	−0.02 (0.06)	0.12 (0.07)	0.12 (0.09)
Psychiatric setting^f^	0.10 (0.15)	−0.10 (0.12)	−0.04 (0.14)	−0.02 (0.16)
Diagnosis^g^	−0.46 (0.18)**	−0.51 (0.12)**	0.06 (0.16)	−0.38 (0.18)*
Psychopathological symptoms	−0.01 (0.08)	−0.16 (0.07)*	−0.06 (0.08)	−0.12 (0.09)
*R* ^*2*^	0.14**	0.56**	0.46**	0.14**

Standardized regression coefficients (beta) with standard errors (SEs) in parentheses are presented

^a^0 = female, 1 = male

^b^Secondary = reference category

^c^0 = non-married (including separated/divorced, widowed and never married), 1 = married (including by common law)

^d^0 = living with someone, 1 = living alone

^e^0 = unemployed, 1 = employed

^f^0 = inpatient ward, 1 = other (including day ward, outpatient clinic and community mental health center)

^g^0 = schizophrenia, 1 = affective disorders

* *p* < 0.05, ** *p* < 0.01

**Table 4:** Bootstrapped point estimates with standard errors and 95% confidence intervals for all indirect effects and the pairwise contrasts of the indirect effects between internalized stigma and QoL

	(Path)	Point estimate	SE	Bootstrapping 95% CI	
				Lower	Upper
Indirect effects					
Via self-esteem	(*a*_1_*b*_1_)	−0.11	0.03	−0.18	−0.05
Via SOC	(*a*_2_*b*_2_)	−0.06	0.03	−0.12	−0.02
Via self-esteem and SOC	(*a*_1_*d*_21_*b*_2_)	−0.06	0.02	−0.11	−0.03
Total indirect effect	–	−0.23	0.04	−0.32	−0.15
Pairwise contrasts					
Self-esteem versus self-esteem and SOC	–	−0.05	0.04	−0.13	0.02
Self-esteem versus SOC	–	−0.05	0.05	−0.15	0.05
Self-esteem and SOC versus SOC	–	0.00	0.03	−0.05	0.05

*SE* standard error, *CI* confidence interval

If the CI does not include zero, the effect is statistically significant at *p* < 0.05

After accounting for socio-demographic and clinical factors, all individual paths between the key variables in the model turned out to be significant, with the exception of the direct effect of internalized stigma on QoL when controlling for the effects of the mediators (i.e., self-esteem and SOC). There was a significant sequential indirect effect of internalized stigma on QoL through self-esteem and SOC [path a_1_d_21_b_2_: beta = –0.06, SE = 0.02; 95% CI (−0.11, −0.03)]. Also significant were simple mediation paths from internalized stigma to QoL via self-esteem [path a_1_b_1_: beta = –0.11, SE = 0.03; 95% CI (−0.18, –0.05)] and from internalized stigma to QoL through SOC [path a_2_b_2_: beta = −0.06, SE = 0.03; 95% CI (−0.12, −0.02)]. Examination of the pairwise contrasts of the indirect effects (see Table 4) revealed that the three indirect effects cannot be distinguished in terms of magnitude (zero is contained in the interval).

## Discussion

In this study, we found evidence for a theoretical model proposing that self-esteem and SOC sequentially mediate the relationship between internalized stigma and QoL among people with mental illness. Our findings are in keeping with previous research which has already convincingly demonstrated a robust association of self-stigma with diminished self-esteem [2, 4] and documented the role of self-esteem in mediating the negative effect of self-stigma on QoL [5, 6]. However, the study results also make an additional contribution to the existing literature by identifying SOC as another important element in the chain of self-stigma consequences and speaking in favor of the idea that self-esteem is a potential GRR for SOC.

It should be emphasized that apart from the two-mediator path, two simple mediation paths with either self-esteem or SOC as single mediators between internalized stigma and QoL were also found to be significant and the three indirect effects did not differ in terms of magnitude. This further corroborates the importance of both of these self-related variables for the QoL and highlights the complexity of the ways in which self-stigma acts on people with mental illness.

Contrary to self-esteem, SOC is a construct that has not been thus far extensively studied in the context of internalized mental health stigma and the harms it causes. This may be somewhat surprising given the well-described role of SOC in coping with stressors or enhancing health and QoL [13, 24, 25] as well as its clear relevance for mental health treatment and rehabilitation [26–28]. Identifying the mediating effects of SOC is noteworthy as it indicates that the mechanism through which self-stigma affects the QoL of people with mental illness involves the restriction of their capacity to manage stress. Thus, for service users, self-stigma not only is a stressor itself, but also a factor diminishing their ability to mobilize the GRRs and to adapt to stressful situations. Given the harmful effects of stress on the course and outcome of mental disorders [29] and the accumulated evidence that stronger SOC is associated with better health (especially mental health) [24], it can be recommended that future studies investigate whether SOC acts as a mediator in the relationship of internalized stigma not only with QoL, but also with various indicators of health and disability in people receiving psychiatric treatment. This could shed more light on the routes through which self-stigma hinders recovery from mental illness.

Regarding implications for clinical practice, with replication this study may support the need for targeting self-esteem and SOC in therapeutic programs aiming to prevent the harmful impact of internalized stigma on people with mental illness. There is some empirical evidence indicating that these personal resources are modifiable by means of specific group interventions. An example of a method of improving self-esteem may be the “self-esteem module” designed by Lecomte et al. [30]. This is a 12-week structured group intervention, consisting of 24 1-h sessions and divided into five blocks addressing the following key aspects of self-esteem: a sense of security, a sense of identity, a sense of belonging, a sense of purpose, and a sense of competence. A randomized cross-over study by Borras et al. [31] has confirmed the effectiveness of this approach among individuals with severe mental disorders, in particular those receiving case-management care. In turn, an example of a promising intervention promoting SOC is a talk-therapy group program developed by Langeland et al. [27, 32]. It consists of 16 weekly group meetings and homework, with mental health professionals acting as group leaders. Its main purpose is to raise participants’ awareness of their potential, their internal and external resistance resources (such as personal qualities, coping abilities and social support), and their ability to use them, and thus to improve their SOC, coping, and level of mental health. In a randomized controlled trial, this intervention was demonstrated to have a significant positive influence on the SOC of people with mental health problems [32]. In the light of the findings obtained, these types of interventions may have the potential to break the chain of negative effects triggered by self-stigma and leading to the impairment of QoL and may be a useful addition to the interventions directly targeting self-stigmatizing beliefs and attitudes, e.g., through psychoeducation or cognitive behavioral therapy (CBT) techniques [33, 34].

Some limitations of the study are to be mentioned. Most importantly, the cross-sectional design does not allow us to draw any definite conclusions about causality. While there is considerable evidence for the detrimental effect of self-stigma on self-esteem [2], it has also been suggested that enhancing self-esteem may lead to the reduction of self-stigma [33]. Similarly, although it seems reasonable to hypothesize that stigma may weaken SOC, alternative models have also been proposed in which SOC is a predictor of stigma [35, 36]. Clearly, longitudinal studies are needed to further disentangle the complex relationships between internalized stigma, self-esteem, SOC, and QoL. Next, the generalizability of the findings may be restricted by the fact that our participants were a convenience sample recruited from just one psychiatric institution. Furthermore, we cannot rule out the possibility that some unmeasured factors account for the relationships observed in this study. It needs to be noted as well that the stigma instrument utilized in the analyses (the SES) is not a pure measure of internalized stigma. In this respect, however, it is similar to the Internalized Stigma of Mental Illness (ISMI) scale [37], the instrument extensively used for assessing internalized stigma among people with mental illness, which is also a multidimensional, complex measure covering several stigma domains (including, e.g., experienced discrimination). Finally, the validation of the SES against other, more fully tested psychometrically internalized stigma instruments is yet to be performed in order to confirm its measurement properties.

Despite these limitations, this research provides further evidence for the significance of internalized stigma as a barrier to recovery from mental illness and extends the understanding of the mechanism of its impact on service users by showing how it may compromise their QoL through undermining their personal resources (such as self-esteem and SOC).
